# Cardiac phase detection in echocardiography using convolutional neural networks

**DOI:** 10.1038/s41598-023-36047-x

**Published:** 2023-06-01

**Authors:** Moomal Farhad, Mohammad Mehedy Masud, Azam Beg, Amir Ahmad, Luai A. Ahmed, Sehar Memon

**Affiliations:** 1 College of Information Technology, United Arab Emirates University, Al Ain, P.O. Box 15551, United Arab Emirates; 2 Institute of Public Health, College of Medicine and Health Sciences, United Arab Emirates University, Al Ain, United Arab Emirates; 3 Indus Medical College, Hyderabad, Pakistan

**Keywords:** Cardiology, Health care

## Abstract

Echocardiography is a commonly used and cost-effective test to assess heart conditions. During the test, cardiologists and technicians observe two cardiac phases—end-systolic (ES) and end-diastolic (ED)—which are critical for calculating heart chamber size and ejection fraction. However, non-essential frames called Non-ESED frames may appear between these phases. Currently, technicians or cardiologists manually detect these phases, which is time-consuming and prone to errors. To address this, an automated and efficient technique is needed to accurately detect cardiac phases and minimize diagnostic errors. In this paper, we propose a deep learning model called DeepPhase to assist cardiology personnel. Our convolutional neural network (CNN) learns from echocardiography images to identify the ES, ED, and Non-ESED phases without the need for left ventricle segmentation or electrocardiograms. We evaluate our model on three echocardiography image datasets, including the CAMUS dataset, the EchoNet Dynamic dataset, and a new dataset we collected from a cardiac hospital (CardiacPhase). Our model outperforms existing techniques, achieving 0.96 and 0.82 area under the curve (AUC) on the CAMUS and CardiacPhase datasets, respectively. We also propose a novel cropping technique to enhance the model’s performance and ensure its relevance to real-world scenarios for ES, ED, and Non ES-ED classification.

## Introduction

Cardiovascular disease is a major cause of death worldwide, accounting for 43.8% of all deaths annually, particularly in individuals with low socioeconomic status^1^. Early diagnosis is critical for effective risk factor management and treatment. Several tests can be performed for cardiac examination, including cardiac magnetic resonance imaging (MRI), echocardiography, and computed tomography (CT). While MRI and CT provide high-quality images^2^, echocardiography is the most affordable, widely available, and noninvasive technique with a low acquisition time^3^. Transthoracic echocardiography is the most commonly used method, and it can diagnose several heart diseases.

**Figure 1:**
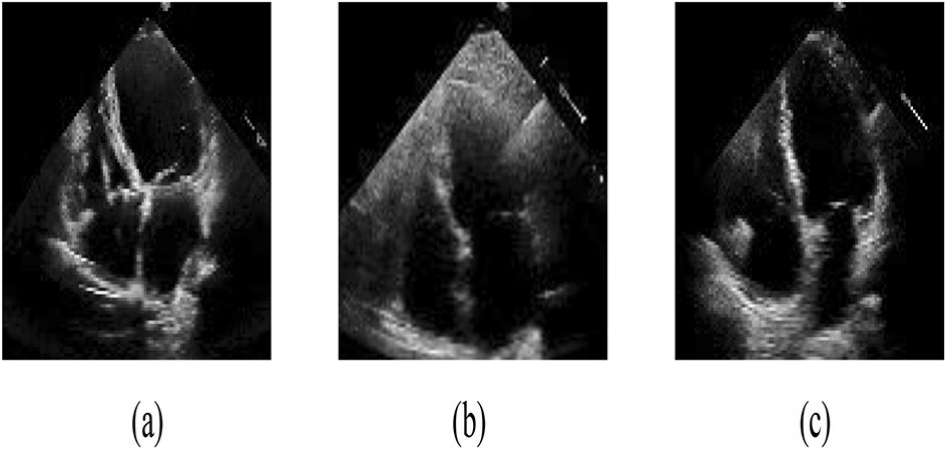
Echocardiograms showing (**a**) ES phase (**b**) ED phase (**c**) Non-ESED frame.

The cardiac cycle is divided into different phases, including isovolumetric contraction and ventricular diastolic filling in the first phase, and isovolumetric relaxation and blood ejection in the second phase^4^. Accurate identification of the end-diastolic (ED) and end-systolic (ES) phases is crucial for measuring heart function and chamber size, which help calculate the ejection fraction^5^, global longitudinal strain, wall thickness, and stroke volume^6^. However, there are some frames in the echocardiography modality that do not represent either ES or ED phases, which we refer to as Non-ESED frames. Identifying ES and ED phases from a small echocardiography sequence, which consists of ES, ED, and Non-ESED images, can be time-consuming and highly dependent on the interpreter’s expertise (Fig. 1).

Research has shown that overworked cardiologists may misdiagnose cardiovascular diseases. According to Gene et al.’s^7^ study in 2017, 39% of patients with acute myocardial infarction were misdiagnosed by doctors. Burnout syndrome, which affects over 50% of cardiologists in the US, can cause stress and reduce concentration, leading to missed diagnoses^8^. In echocardiography, accurately identifying end-diastolic (ED) and end-systolic (ES) phases is essential for assessing heart function and chamber size. Traditionally, LV segmentation-based methods^9,10^ have been used to identify the ES and ED frames based on the assumption that the smallest and largest LV segmented cross-sections in a cardiac cycle represent the ES and ED frames, respectively. However, these methods require complex preprocessing and are prone to errors due to the noisy nature of echocardiograms. Furthermore, they typically require initialization from user input using active contours and deformable models, which can be time-consuming and impractical for real-world applications. Researchers have also used various image-processing techniques, such as velocity spectrum envelopes^11^ and Gaussian mixture techniques^1^ , for automatic interpretation. However, deep learning techniques^12^, which learn and recognize patterns from data, provide better accuracy and require minimal preprocessing. In this paper, the authors use a convolutional neural network for identifying ES and ED frames in echocardiography images.

The focus of this research is on developing a method for automatically classifying the ED, ES and Non-ESED phases in echocardiography imaging without the need for manual measurement or automated segmentation of the left ventricle. The approach is named DeepPhase, which stands for “Deep learning based cardiac Phase detection using echocardiography imaging”. This is the first attempt to detect phases using this approach. The contributions of this study include proposing an effective CNN model for the classification tasks, introducing a custom loss function that enhances the prediction accuracy of the CNN model, providing a new dataset annotated by two expert cardiologists, demonstrating the efficacy of the approach through experiments on benchmark and newly introduced datasets, and introducing a cropping technique to improve performance on the three-phase classification task. The effectiveness of the DeepPhase model was found to be better than relevant state-of-the-art techniques.

## Related work

In this section, we review previous studies that explored computer vision, classical machine learning, and DL techniques for the classification and segmentation of echocardiography imaging. We found that most of the work related to echocardiography images focused on two primary purposes: segmentation of the left ventricle (LV) and classification of cardiac views and phases.

### Computer vision algorithms

Guo et al.^13^ treated the LV segmentation task as a smoothing problem; accordingly, they proposed an adaptive sparse smoothing technique for LV segmentation from echocardiograms and compared the performance of their method with that of the active contour method. To segment an anterior mitral leaflet, Sultan et al.^14^ proposed sampling of echocardiographic videos over time by exploring a partly automated evaluation of a scanning line, which provided a new image space as an output, referred to as the Virtual M-mode. However, this method was not fully automatic because to initialize the Virtual M-mode, a doctor needed to manually specify a single point for the posterior wall of the aorta. The recall and precision values of 0.94 and 0.50, respectively, were achieved. However, segmentation oversizing was the limitation of the proposed method, typically caused by improper scanning.

### Classical machine learning algorithms

Melo et al.^15^ developed a framework to detect the posterior wall of the LV from echocardiography images by merging a light gradient boosting machine with morphological features considering the PLAX view. They achieved 67% sensitivity and 98% specificity. Li et al.^16^ proposed a method to realize the exact segmentation of myocardium in echocardiography. This method combined boundary box detection, a statistical shape model, and a random forest algorithm and could be applied to nonmedical images containing large intensity variations. They achieved a standard deviation of 1.43. In addition, Moghaddasi and Nourian^17^ proposed a model to categorize the severity of mitral regurgitation disease, in which the features were first extracted using the local binary pattern. Then, a support vector machine was used to grade the videos. The dataset considered in this study comprised 102 videos, and 99% accuracy was obtained. Ghori et al.^1^ proposed an echocardiography view classification method that employed a Gaussian mixture model and supports a vector machine. They introduced the ECHO 1.0 dataset, which contained 637 echocardiography videos. They achieved an accuracy of 90.8%.

### Deep learning algorithms

The section titled “Deep learning algorithms” discusses various studies that have employed deep learning (DL) techniques for echocardiography image analysis. Leclerc et al.^12^ utilized multiple DL models, including UNet, UNet++, and stacked hourglasses, to segment the left ventricular (LV) endocardium and myocardium using the Cardiac Acquisitions for Multistructure Ultrasound Segmentation (CAMUS) dataset. Similarly, Madani et al.^18^ applied a semi-supervised DL approach, which combined a convolutional neural network (CNN), generative adversarial networks, and UNet, to classify echocardiographic views and segment LV hypertrophy. Nizar et al.^19^ employed faster region-based convolutional neural networks and a single-shot multibox detector for aortic valve segmentation, while Moradi et al. used UNet and feature pyramid network on the CAMUS dataset for LV segmentation. Barcaro et al.^9^ used Otsu’s threshold for LV segmentation and ED and ES frame classification for computing LV ejection fraction. Lane et al.^20^ formulated ES and ED phase detection as a regression problem using a CNN-based model, and Wegner et al.^21^ used a CNN based on VGG19 for view classification from 2D echocardiography. Litjens et al.^22^ identified lack of uniform performance evaluation and annotated data as the main challenges in DL-based echocardiography research and suggested involving physicians and medical staff in identifying areas where automation is required. Finally, Dezaki et al.^23^ explored DL techniques for ED and ES phase detection from four-chamber view echocardiography cine series, using a custom loss function based on mean squared error and a combination of CNN and recurrent neural network modules. The current study is motivated by Dezaki et al.’s^23^ work, but it differs in several aspects, such as using echocardiography images instead of cine series, and formulating ES and ED detection as a classification problem instead of a regression problem. The study justifies the use of echo images due to their efficient storage and transmission, as well as their suitability for accurate diagnosis by cardiologists and computer systems.

This study is expected to provide a foundation for achieving impressive results for cardiac phase detection in the apical two-chamber view and apical four chamber view. Unlike previous research, we carried out the task without LV segmentation, using only a custom CNN architecture and a dataset of 1000 images.

## Methodology

This section is divided into two subsections. We discussed the ES and ED phase classification methodology in the first subsection. Afterwards, we discussed the methodology for ES, ED and Non-ESED frames classification.

### ES and ED classification

**Figure 2:**
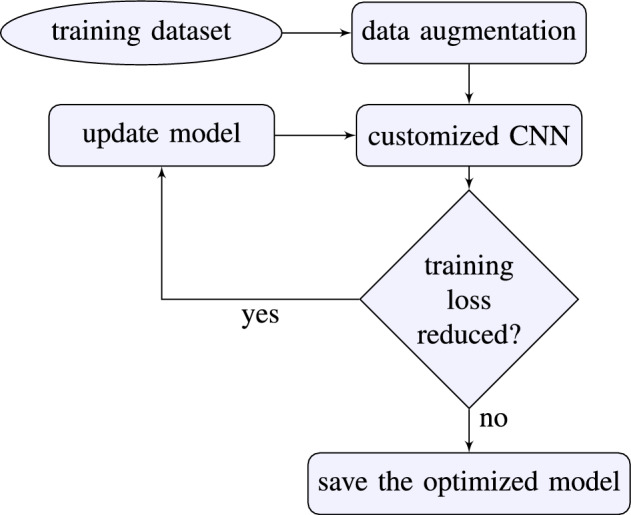
Phase I: model training.

**Figure 3:**
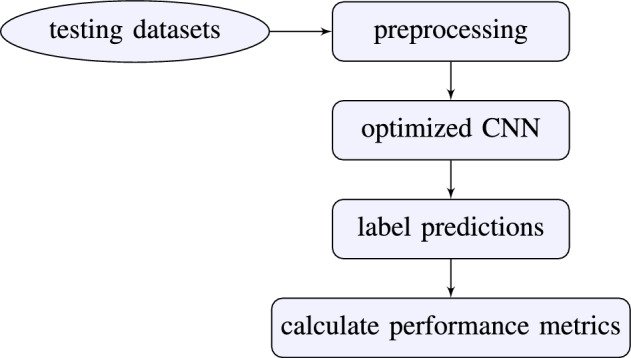
Phase II: model testing.

We divided the methodology into two phases: model training and model testing. Each phase has several substages, e.g., preprocessing and data augmentation, optimizing and testing the DeepPhase model, and calculating results^24^. The high level architecture of DeepPhase is given in Figs.2 and 3.

**Figure 4:**
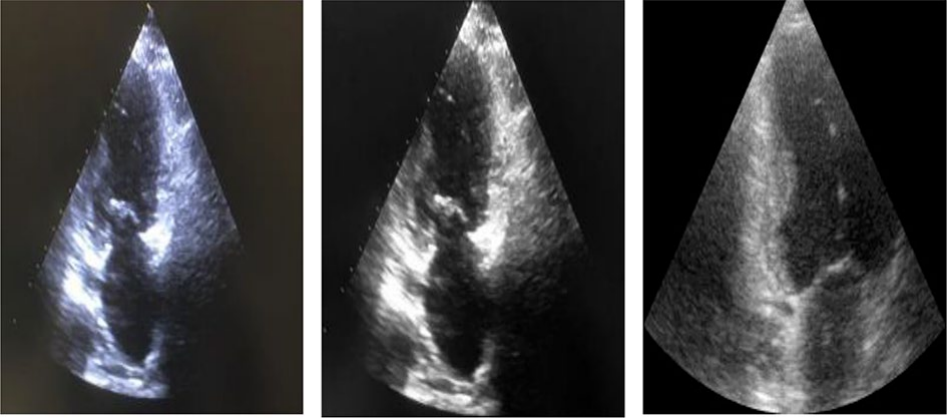
Left: an unprocessed image from CardioPhases dataset; middle: the same image after applying to preprocess; right: an image from the CAMUS dataset.

#### Phase I: model training

##### Training dataset

The DeepPhase model was trained and validated using the CAMUS dataset, which was developed by Leclerc et al. in 2019^12^. The lack of comprehensive and publicly available data for echocardiography motivated the creation of the CAMUS dataset. It includes the reports of 500 patients with ED and ES frames in four-chamber and two-chamber views, acquired from the St. Etienne hospital in France using GE Vivid E95 ultrasound scanners with a GE M5S probe. The dataset comprises good-, poor-, and medium-quality images, and labelling was performed by three cardiologists.

##### Data augmentation

To improve the generalization of the CNN network and reduce overfitting, data augmentation was used. Multiple variants of each training image were generated by varying augmentation hyperparameters across specified ranges. Four augmentation methods were employed:

(i) *Rescale* Pixel values in gray-scale images range from 0 to 255. To transform every pixel value from the range $\left[ 0,255 \right] \rightarrow \left[ 0,1 \right]$, a rescale factor of 1/255 was used.

(ii) *Shear* A shear intensity of 0.2 was set to distort the image along an axis and rectify or create the perception angles. This augmentation method models how human beings see things from different angles.

(iii) *Zoom* A zoom range of 0.2 was set to randomly zoom the image and add new pixels.

(iv) *Horizontal flip* The augmented images were generated by flipping the original image horizontally.

##### DeepPhase model

**Figure 5:**
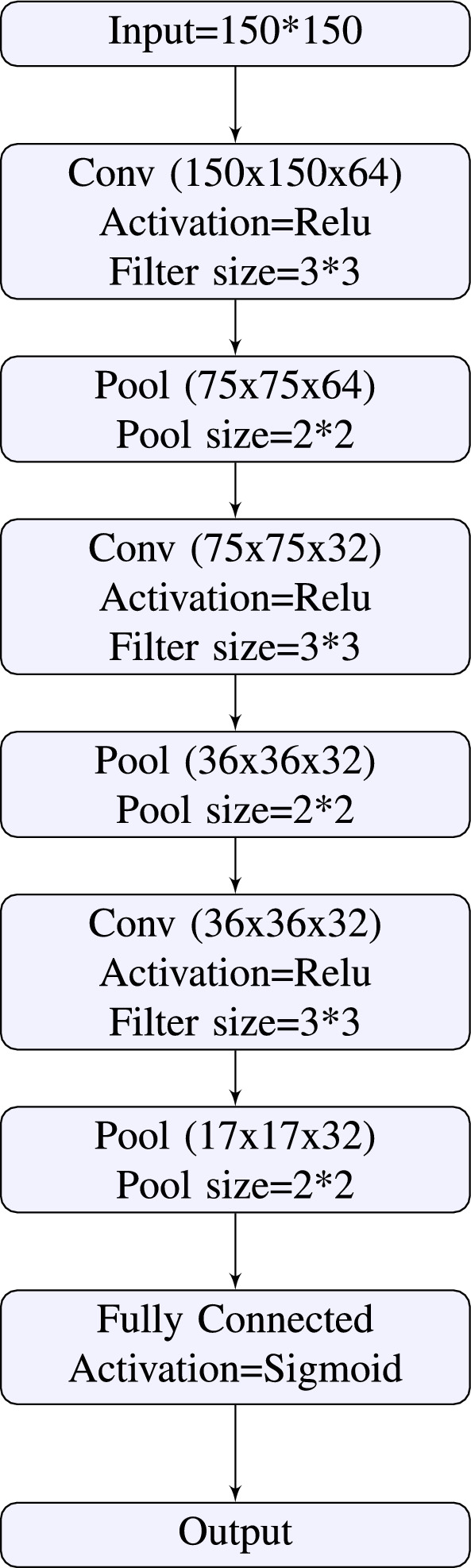
Architecture of the DeepPhase.

A typical CNN architecture consists of convolutional, pooling, and fully connected layers^25^. In this study, we propose a 9-layer CNN architecture, including input, convolutional, pooling, fully connected, and output layers, specifically designed for cardiac phase detection^24^. The CNN architecture consists of three convolutional layers, three pooling layers, and a single fully connected layer. Each convolutional layer applies a (3,3) convolution kernel to the input to generate feature maps. The rectified linear unit (ReLU) is used as the activation function to prevent vanishing gradients during backpropagation and introduce nonlinearity to the convolution layer output. Figure 5 shows the detailed structure of the DeepPhase architecture.

##### Model optimization

Model optimization involves hyperparameter selection and loss function construction, which are described below.

(i) *Hyperparameter selection* The hyperparameter settings are described in “Parameter configuration” and shown in Fig. 5.

(ii) *Loss function* The loss function approximates the model’s loss so that the weights can be adjusted to reduce the loss in the next epoch. To reduce training loss, we employed regularization techniques that efficiently decrease the model’s variance without significantly increasing its bias. The proposed loss function is based on the mean squared error (MSE) and consists of two parts represented by Eqs. (1) and (2).

1 $$\begin{aligned} MSE(\chi ,M)= & {} \frac{1}{n} \sum _{i=1}^n (\tau (x_i)-M(x_i) )^2 \end{aligned}$$

2 $$\begin{aligned} mean (\chi ,M)= & {} \frac{1}{n} \sum _{i=1}^n (\tau (x_i)-M(x_i) ) \end{aligned}$$

where $\chi$ is the set of n instances ${(x_1,x_2,\ldots ,x_n)}$ and M represents the prediction model. In addition, $\tau (x_i)$ is the actual label of $x_i$, where $\tau (x_i)\in {(0,1)}$, $M(x_i)$ represents the label of $x_i$ predicted by the model, and n is the total number of instances. The proposed loss function is given below.

3 $$\begin{aligned} L(\chi ,M) = MSE(\chi ,M)+\beta .mean(\chi ,M) \end{aligned}$$

where $L(\chi ,M)$ is the loss function. The formula in Eq. (3) indicates that after taking the mean difference between the true and predicted labels, the number is multiplied by a hyperparameter $\beta>$0. The result is then added to $MSE(\chi ,M)$; this adds an additional term to the traditional MSE loss function, which further penalizes the model for incorrect classifications. We have empirically shown the effectiveness of the proposed loss function over MSE and other traditional loss functions in “Experiments”.

#### Phase II: model testing

##### Datasets

In this research, the DeepPhase model’s performance was evaluated on two datasets. The first dataset consisted of 100 images from the CAMUS dataset, which was already partitioned by the dataset authors. The second dataset, called the CardiacPhase dataset, was fully annotated and developed specifically for this study. It contains echocardiograms from 35 patients who were treated at the CardioLife diagnostic center in Hyderabad, Pakistan. No specific data selection method was used to ensure clinical realism, resulting in a highly heterogeneous dataset of both pathological cases and image quality, typical of routine clinical practice data. The dataset comprised 70 images and was used as a testing dataset to evaluate the DeepPhase model’s performance. The echocardiography data were obtained between June and September 2020, and two cardiologists annotated the data in this dataset. The data were captured in the ED and ES phases in the two-chamber view for each patient. The data collection was performed following the principles of the Declaration of Helsinki.

To ensure that the images from both datasets appeared similar, several preprocessing techniques were applied because they were obtained using different scanners, probes, and protocols. The images were cropped to eliminate unwanted labels and lines, and the image size was adjusted to 150 x 150 x 1, and the images were denoised using linear and mean filters. The cropping process was necessary to ensure data anonymity. Figure 4 displays three frames, all showing the ED phase of the cardiac cycle, from the two datasets. The first frame shows an unprocessed image from the CardiacPhase dataset, the second frame displays the same image after post-processing, and the final frame presents an image from the CAMUS dataset.

### ES, ED and non-ESED classification

**Figure 6:**
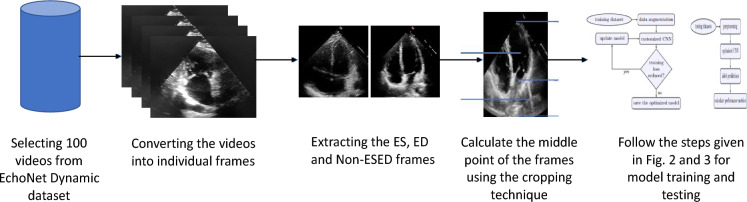
The methodology followed for ES, ED and Non-ESED classification.

This section discusses the methodology followed for ES, ED and Non-ESED classification. The overall process, shown in Fig. 6, is explained in the following subsections.

#### EchoNet dynamic dataset

Ouyang et al.^26^ created the EchoNet-Dynamic dataset containing 10,030 echocardiography videos covering a variety of typical lab imaging acquisition conditions. All images have labelled measurements, including LV volume at end-systole and end-diastole, ejection fraction and expert tracings of the left ventricle. These measurements are obtained by a registered sonographer and verified by a level 3 echocardiographer. The dataset contains apical-4-chamber echocardiography videos from patients who underwent echocardiography tests between 2016 and 2018 at Stanford University Hospital. Each video was cropped to delete the text and information outside of the scanning sector, and the resulting images were downsampled into standardized 112x112 pixel videos.

#### Creation of dataset for ES, ED and non-ESED frames classification

We have randomly selected 60 videos from training, 20 videos from validation and 20 videos from the test set of the EchoNet Dynamic dataset. We have chosen three frames from each video, i.e., ES, ED and Non-ESED. The ES and ED frames are labelled by the authors^26^ for each video, and the Non-ESED frame is chosen randomly from the rest of the frames.

#### The cropping technique

**Figure 7:**
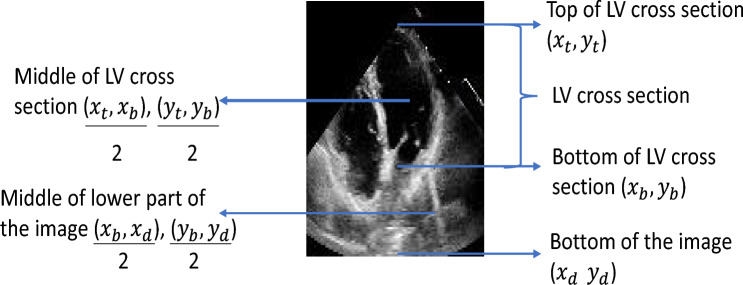
A frame from EchoNet dynamic dataset.

**Figure 8:**
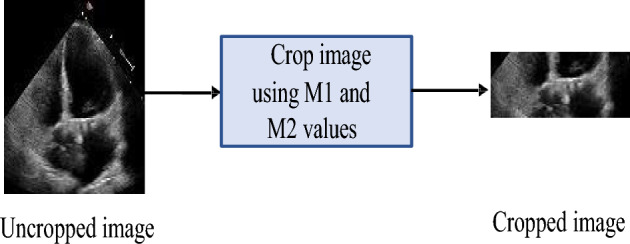
The cropping technique.

To improve the performance of the model, we cropped all the frames to enable the model to extract the most significant features from the frames. We clipped the images so that only the section which helps differentiate between ES, ED and Non-ESED frames is fed to the model. We utilized the metadata provided with the ‘EchoNet Dynamic’ dataset to crop the images. The metadata contains tracings of LV at the endocardial border. The expert tracings are represented by a collection of paired coordinates corresponding to each human tracing. The initial pair of coordinates show the direction and length of the long axis of the LV as represented by a vertical line in Fig. 7. The remaining coordinate pairs represent short axis linear distances starting from the apex of the heart to the mitral apparatus, as shown by a horizontal line in Fig. 7. To crop the images, we averaged the first pair of coordinates to find the average length of LV. Once we got the average length, we calculated the middle point of the LV, which we referred to as $M_1$, using the top and bottom coordinates of the length of the long axis. Next, we calculated the second middle point, which we referred to as $M_2$, from the average of bottom coordinates of the LV to the end of the image as given in Eqs. (4) and (5). The resulting image is shown in Fig. 8.

4 $$\begin{aligned}{} & {} M_1 \rightarrow \text {mean of the middle of LV cross section} \rightarrow (X_1, Y_1) \end{aligned}$$

5 $$\begin{aligned}{} & {} M_2 \rightarrow \text {mean of the middle of the lower part of the image} \rightarrow (X_2, Y_2) \end{aligned}$$

Let $(x_{t}^{i},y_{t}^{i})$ represent the top of the LV, $(x_{b}^{i},y_{b}^{i})$ represent the bottom of the LV, and $(x_{d}^{i},y_{d}^{i})$ represent the bottom of the image. Equations (6), (7), (8), and (9) are used to find $X_1, Y_1, X_2$, and $Y_2$, where *n* is the total number of images.

6 $$\begin{aligned} X_1= & {} \frac{1}{n}\sum _{i=1}^{n}\frac{\left( x_{i}^{t}+x_{i}^{b}\right) }{2} \end{aligned}$$

7 $$\begin{aligned} Y_1= & {} \frac{1}{n}\sum _{i=1}^{n}\frac{\left( y_{i}^{t}+y_{i}^{b}\right) }{2} \end{aligned}$$

8 $$\begin{aligned} X_2= & {} \frac{1}{n}\sum _{i=1}^{n}\frac{\left( x_{i}^{b}+x_{i}^{d}\right) }{2} \end{aligned}$$

9 $$\begin{aligned} Y_2= & {} \frac{1}{n}\sum _{i=1}^{n}\frac{\left( y_{i}^{b}+y_{i}^{d}\right) }{2} \end{aligned}$$

## Experiments

### Datasets

We used the CAMUS, CardiacPhase and EchoNet Dynamic datasets (Discussed in “Methodology”).

### Competing approaches

In this study, we evaluated and compared the following competing approaches.

#### DeepPhase

The proposed method comprises a CNN and the custom loss function. The architecture of the DeepPhase model is shown in Fig. 5, and the custom loss function is described in Methodology.

#### CNN Proposed by Siddiqi^27^

The DeepPhase model was compared with Siddiqi’s^27^ model, which was used to diagnose pneumonia from X-ray images. Although Dezaki et al.^6^ work is relevant to ours, as discussed in “Related work”, it is not directly comparable to DeepPhase. Firstly, they used cine series of echocardiography imaging, and secondly, they dealt with the problem as regression and not classification.

The key differences between the DeepPhase model and Siddiqi’s^27^ CNN model are summarized below. In Siddiqi’s^27^ proposed architecture, a pair of convolution layers are inserted at the start and after every dropout layer. By contrast, the DeepPhase model utilizes a single convolution layer at the start and after every pooling layer to reduce resource consumption, e.g., storage and computation time. Further, a pooling layer is employed after each pair of convolutional layers in the architecture proposed by Siddiqi^27^. We employed max pooling in the DeepPhase model because this technique is superior in selecting invariant features; thus, generalizability is improved. The max pooling operation also converges significantly faster during training (compared with other pooling operations). Here, the most frequently used configuration for pooling layers was a pool size of (2, 2). With these settings, each pooling layer discarded 75% of the activations. Siddiqi’s^27^ proposed architecture uses a dropout layer (with a dropout rate of 0.2) after each pooling layer. In our proposed architecture, we did not use dropout layers. The primary purpose of a dropout layer is to prevent the model from overfitting. We employed several techniques to avoid overfitting in the DeepPhase model, e.g., the regularization of the loss function and data augmentation. Finally, we employed a single dense layer. By contrast, Siddiqi’s model^27^ uses two dense layers.

#### Model proposed by Lane et al.^20^

We have compared the performance of DeepPhase model for ES, ED and Non-ESED classes with the model proposed by Lane et al.^20^. The researchers performed ES and ED detection by combining CNN and LSTM model on EchoNet dataset.

#### Model proposed by Wegner et al.^21^

Wegner et al. proposed a model for view classification from 2D echocardiography. Their model consists of CNN using the VGG19 as a base model.

#### Pretrained models

We have evaluated the performance of the DeepPhase model with pretrained models such as VGG16, ResNet, EfficientNet and ModeileNet.

#### Other traditional loss functions

(i) *DeepPhase-MAE* The DeepPhase model uses the mean absolute error (MAE) loss function (instead of the custom loss function).
(ii) *DeepPhase-CrossEntropy* The DeepPhase model uses the cross entropy loss function (instead of the custom loss function).
(iii) *DeepPhase-MSE* The DeepPhase model uses the mean squared error (MSE) loss function (instead of the custom loss function).
(iv) *DeepPhase-L1 Regularizer* We set the value of 0.1 for L1 regularizer and train it with MSE loss function. These parameters, namely the value of 0.1 for L1 regularizer and training with MSE loss function, were chosen based on their superior performance compared to other parameters. The decision was made after evaluating the competing models and their corresponding results, as shown in Table 6.
(v) *DeepPhase-L2 Regularizer* In this experiment, we set the value of 0.1 for L2 regularizer and train it with MSE loss function.

### Parameter configuration

The publishing authors divided the CAMUS dataset into training and testing sets. There are 900 frames or images (two images per patient) in the training set and 100 frames in the testing set. From the 900 images of the training set, we allocated 90 images as a validation set. The validation set helps avoid overfitting and assists in parameter selection. The model was optimized for other hyperparameters such as learning rate, batch size, and optimizer using the randomized grid search method. Here, the Adam optimizer was used with a batch size of 10 and a learning rate of 0.00001.

### Hardware and software configuration

The compared models were coded in Python (version 3.6.5; 64-bit) using the open-source Keras (version 2.1.5) library as the backend and open-source TensorFlow (version 2.5.0) library for the CNN algorithms. The models were executed using the GPU runtime setting of Google Colab on the Google cloud.

**Table 1:** Comparison of DeepPhase, Siddiqi’s CNN and pre-trained models performance.

	CAMUS				CardiacPhase			
	AUC	Loss	Recall	Precision	AUC	Loss	Recall	Precision
VGG-16	0.84	0.18	0.78	0.72	0.79	0.25	0.72	0.7
ResNet	0.92	0.16	0.78	0.72	0.61	0.75	0.6	0.6
EfficientNet	0.86	0.25	0.76	0.7	0.72	0.21	0.7	0.68
MobileNet	0.91	0.17	0.76	0.7	0.8	0.22	0.75	0.72
CNN proposed by Siddiqi^27^	0.7	0.31	0.76	0.69	0.61	0.83	0.72	0.63
DeepPhase	**0**.**96**	0.08	0.8	0.74	**0**.**82**	0.14	0.85	0.8

Significant values are in bold.

### Evaluation metrics

The performance of each model in all experiments was evaluated based on the area under the receiver operating characteristic curve (AUC) and loss.

## Evaluation

We have divided this section into two parts. Initially, we discussed the performance of the DeepPhase model for ES and ED classification^24^ and then evaluated the model’s performance for ES, ED and Non-ESED cardiac phases.

### Evaluation of the DeepPhase model performance on ES and ED cardiac phases

We have compared the performance of the DeepPhase model with relevant state-of-the-art models, and the results are given in the following subsections.

#### Comparison among DeepPhase, Siddiqi’s CNN and pretrained models

The comparisons of each model’s performance are shown in Table 1, demonstrating that the DeepPhase model outperformed Siddiqi’s model^27^ and all four pre-trained models on the CAMUS and CardiacPhase datasets. The performance of ResNet is satisfactory for the CAMUS dataset with an AUC of 0.92, while for the CardiacPhase dataset, VGG-16 achieved an AUC of 0.79. The performance of MobileNet is equally suitable for both CAMUS and CardiacPhase datasets, with the AUC of 0.92 and 0.8, respectively. Siddiqi’s CNN^27^ performed better on the CAMUS dataset than the CardiacPhase dataset, with an AUC of 0.7 and 0.61, respectively.

**Figure 9:**
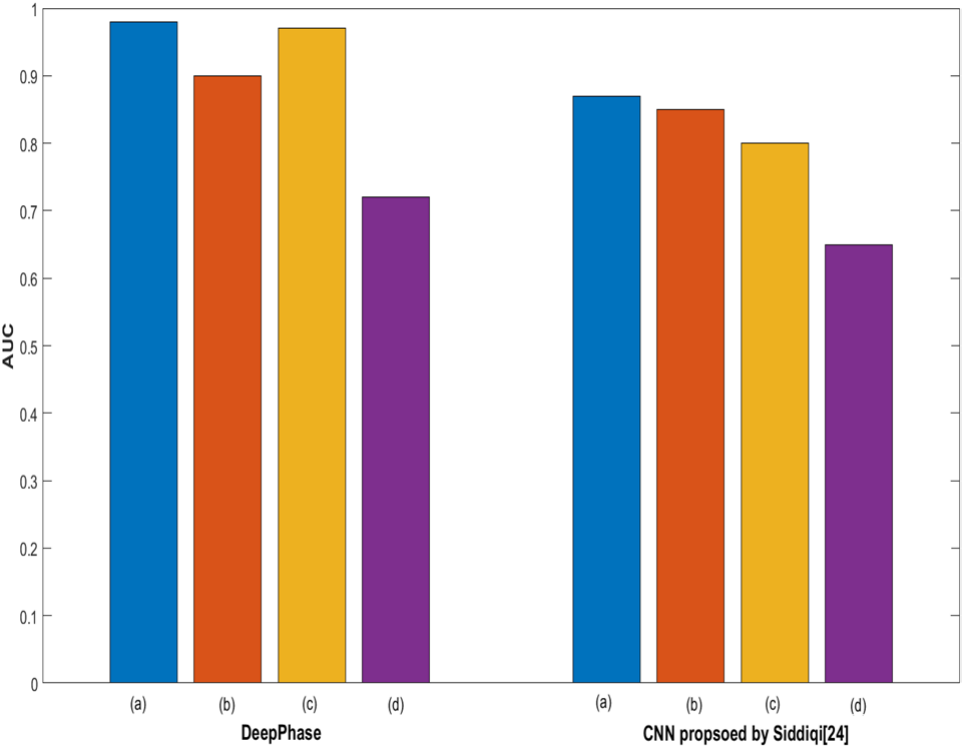
The performance of competing approaches when (**a**) trained and tested over apical four chamber viewpoint (CAMUS) (**b**) when trained over apical two chamber viewpoint (CAMUS) and tested over the apical four-chamber viewpoint of CAMUS dataset (**c**) trained over apical four-chamber viewpoint (CAMUS) and tested over apical two chamber viewpoint (CAMUS) (**d**) when trained over apical four chamber viewpoint (CAMUS) and tested over apical two chamber viewpoint (CardiacPhase).

**Figure 10:**
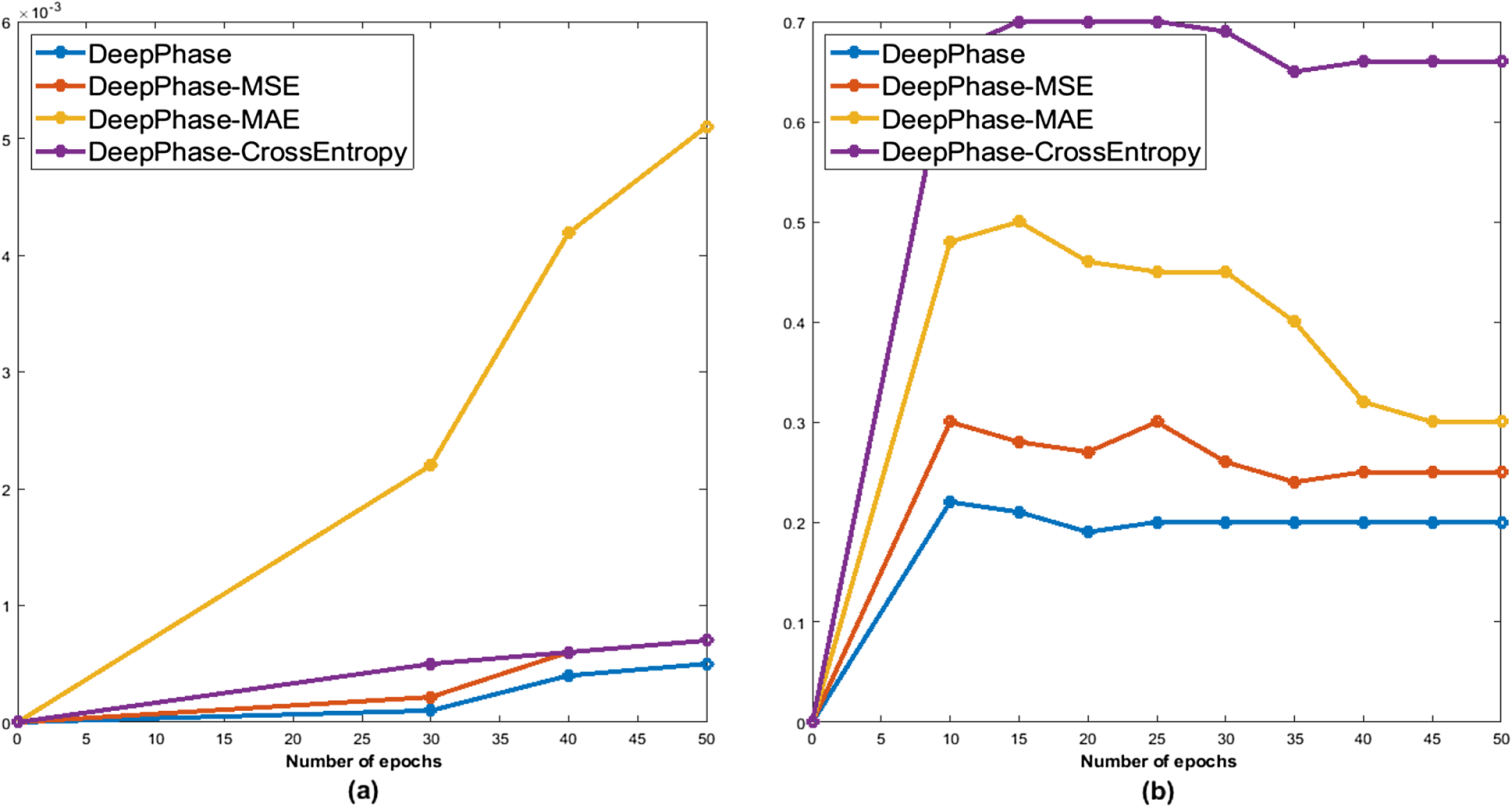
(**a**) Average weights of the DeepPhase model using different loss functions (**b**) Convergence of different loss functions.

**Table 2:** Performance of DeepPhase with different $\beta$ values.

	CAMUS		CardiacPhase	
	AUC	Loss	AUC	Loss
DeepPhase ($\beta$ = 0.05)	0.85	0.18	0.65	0.35
DeepPhase ($\beta$ = 0.1)	0.96	0.08	0.7	0.26
DeepPhase ($\beta$ = 0.15)	0.76	0.22	0.8	0.19
DeepPhase ($\beta$ = 0.2)	0.8	0.14	0.92	0.14
DeepPhase ($\beta$ = 0.3)	0.81	0.18	0.8	0.17
DeepPhase ($\beta$ = 0.4)	0.85	0.16	0.75	0.12
DeepPhase ($\beta$ = 0.5)	0.84	0.17	0.74	0.19
DeepPhase ($\beta$ = 0.6)	0.85	0.12	0.7	0.10

#### Evaluation of parameter sensitivity

In our custom loss function, shown in Eq. (3), $\beta$ played the role of a hyperparameter. Finding the effect of different values of $\beta$ was crucial for the performance of our model. We experimented with different $\beta$ values, as shown in Table 2. It is evident from Table 2 that when $\beta$ value reaches 0.5 and 0.6, the values of the evaluation metrics become undeviating. The best testing AUC was achieved when $\beta$ value was set at 0.1 for the CAMUS dataset and 0.3 for the CardiacPhase dataset. As we kept increasing the value, the evaluation values went down and became static.

**Table 3:** Performance of competing models for ES and non-ESED frames.

Competing approaches	AUC	Loss	Precision	Recall
DeepPhase	0.8	0.15	0.78	0.75
CNN proposed by Siddiqi^27^	0.68	0.27	0.6	0.58
VGG16	0.68	0.27	0.6	0.62
MobileNet	0.7	0.26	0.67	0.68
EfficientNet	0.68	0.25	0.62	0.68
ResNet	0.71	0.28	0.67	0.69
Lane et al.^20^	0.71	0.16	0.72	0.7
Wegner et al.^21^	0.76	0.22	0.7	0.72

**Table 4:** Performance of competing models for ED and non-ESED frames.

Competing approaches	AUC	Loss	Precision	Recall
DeepPhase	0.85	0.15	0.78	0.72
CNN proposed by Siddiqi^27^	0.7	0.31	0.62	0.65
VGG16	0.68	0.27	0.6	0.62
MobileNet	0.74	0.26	0.65	0.68
EfficientNet	0.76	0.25	0.7	0.72
ResNet	0.71	0.28	0.7	0.69
Lane et al.^20^	0.8	0.17	0.7	0.72
Wegner et al.^21^	0.81	0.27	0.71	0.73

**Table 5:** Performance of competing models for ES, ED and Non-ESED frames.

Competing approaches	AUC	Loss	Precision	Recall
DeepPhase	0.79	0.14	0.76	0.78
CNN proposed by Siddiqi^27^	0.72	0.29	0.66	0.68
VGG16	0.58	0.37	0.6	0.6
MobileNet	0.68	0.28	0.61	0.64
EfficientNet	0.76	0.25	0.7	0.72
ResNet	0.71	0.28	0.7	0.69
Lane et al.^20^	0.72	0.29	0.71	0.7
Wegner et al.^21^	0.75	0.32	0.7	0.7

#### Evidence of generalization of DeepPhase

In this section, we carried out the following experiments to prove the generalization of DeepPhase.

##### Trained over tested over different viewpoints

In this experiment, we evaluate the performance of our model when trained with two chambers and tested with four-chamber viewpoints (and vice-versa). We utilized the data from the CAMUS dataset, and hyper-parameters have been adopted from the above apical two chamber view experiments.

The first bar in Fig. 9 shows the results of experiment 01, where the model is trained and tested over the apical four chamber viewpoint of the CAMUS dataset. We have kept the value of $\beta$ = 0.1 as the model performs well on this value (faster convergence and lowest weights). The DeepPhase model achieved the highest AUC of 0.98 at epoch 50, while the model proposed by Siddiqi^27^ achieved the highest AUC of 0.87, respectively. The second bar in Fig. 9 shows experiment 02, where the DeepPhase model is trained over an apical two chamber view and tested over an apical four chamber view of the CAMUS dataset. The model proposed by Siddiqi^27^ achieved the highest AUC of 0.85. The third bar of the graph visualizes experiment 03, where the DeepPhase model is trained over the apical four chamber viewpoint and tested over the apical two chamber viewpoint of the CAMUS dataset. The DeepPhase model achieved the highest AUC of 0.9 with loss = 0.08. The DeepPhase performance declined from 0.97 to 0.9 when trained over an apical two chamber view. It is noticeable here that the DeepPhase model performed consistently well when trained over an apical four chamber view. After learning from this viewpoint, the model successfully identified ES and ED frames from another viewpoint. This observation can be useful when no or less data is available for one of the viewpoints. In experiment 04, we trained the model over the apical four-chamber viewpoint and tested it over the CardiacPhase dataset, which contains apical two chamber ES and ED frames. Afterwards, the fourth bar of the graph shows that the highest AUC achieved by the DeepPhase model is 0.72, while the other model achieved 0.62. All of the above experiments confirmed the performance consistency of the DeepPhase model not only on daily clinical data but also another clinically significant cardiac views.

#### Performance analysis of the custom loss function

To further evaluate the performance of the custom loss function, we performed the following two experiments.

##### Weights

Deep learning models with smaller weights result in more stable training and are less likely to overfit^28^. A model with large weights can signify an unsteady network where small changes in the input can lead to significant changes in the output^29^. This will result in poor performance when predicting new data. Our custom loss function results in smaller weights and a simpler model. Figure 10a shows that the model trained with the custom loss function results in the lowest weights compared to the other models, who are trained using MSE, MAE and binary cross-entropy.

##### Convergence

In order to show the effectiveness of the custom loss function, we compared its convergence rate with other traditionally used loss functions such as MSE, MAE and binary cross-entropy. Figure 10b visualizes the training procedure and convergence of the aforementioned DeepPhase models. From this figure, we observe that the proposed custom loss function converged faster than other counterparts and achieved lower loss values. DeepPhase with $\beta$ = 0.3 converged slower than $\beta$ = 0.1 and 0.2.

**Table 6:** Summary comparison among traditional loss functions and DeepPhase.

Competing methods	CAMUS		CardiacPhase	
	AUC	Loss	AUC	Loss
DeepPhase ($\beta$ = 0.2)	0.8	0.14	**0**.**82**	0.16
DeepPhase ($\beta$ = 0.1)	**0**.**96**	0.08	0.7	0.26
DeepPhase-CrossEntropy	0.58	0.9	0.70	0.94
DeepPhase-MAE	0.71	0.31	0.6	0.79
DeepPhase-MSE	0.72	0.35	0.65	0.32
DeepPhase-L1 Regularizer	0.74	0.32	0.7	0.34
DeepPhase-L2 Regularizer	0.7	0.35	0.68	0.38

Significant values are in bold.

**Table 7:** Performance of competing models for ES and non-ESED frames.

Competing approaches	(Uncropped images)				(Cropped images)			
	AUC	Loss	Precision	Recall	AUC	Loss	Precision	Recall
DeepPhase	0.73	0.2	0.63	0.7	0.8	0.15	0.78	0.75
CNN proposed by Siddiqi^27^	0.45	0.52	0.52	0.5	0.68	0.27	0.6	0.58
VGG16	0.65	0.3	0.56	0.59	0.68	0.27	0.6	0.62
MobileNet	0.67	0.28	0.64	0.65	0.7	0.26	0.67	0.68
EfficientNet	0.64	0.27	0.55	0.6	0.68	0.25	0.62	0.68
ResNet	0.63	0.3	0.5	0.55	0.71	0.28	0.67	0.69
Lane et al.^20^	0.68	0.22	0.6	0.62	0.71	0.16	0.72	0.7
Wegner et al.^21^	0.63	0.22	0.61	0.63	0.76	0.22	0.7	0.72

**Table 8:** Performance of competing models for ED and Non-ESED frames.

Competing approaches	(Uncropped images)				(Cropped images)			
	AUC	Loss	Precision	Recall	AUC	Loss	Precision	Recall
DeepPhase	0.7	0.22	0.64	0.67	0.85	0.15	0.78	0.72
CNN proposed by Siddiqi^27^	0.45	0.52	0.49	0.5	0.7	0.31	0.62	0.65
VGG16	0.63	0.32	0.52	0.56	0.68	0.27	0.6	0.62
MobileNet	0.71	0.28	0.64	0.65	0.74	0.26	0.65	0.68
EfficientNet	0.66	0.27	0.6	0.62	0.76	0.25	0.7	0.72
ResNet	0.63	0.3	0.5	0.55	0.71	0.28	0.7	0.69
Lane et al.^20^	0.7	0.23	0.62	0.68	0.8	0.17	0.7	0.72
Wegner et al.^21^	0.69	0.26	0.6	0.68	0.81	0.27	0.71	0.73

**Table 9:** Performance of competing models for ES, ED and Non-ESED frames.

Competing approaches	(Uncropped images)				(Cropped images)			
	AUC	Loss	Precision	Recall	AUC	Loss	Precision	Recall
DeepPhase	0.72	0.2	0.63	0.7	0.79	0.14	0.76	0.78
CNN proposed by Siddiqi^27^	0.47	0.54	0.52	0.5	0.72	0.29	0.66	0.68
VGG16	0.45	0.38	0.52	0.41	0.58	0.37	0.6	0.6
MobileNet	0.61	0.31	0.6	0.58	0.68	0.28	0.61	0.64
EfficientNet	0.62	0.32	0.6	0.62	0.76	0.25	0.7	0.72
ResNet	0.63	0.3	0.5	0.55	0.71	0.28	0.7	0.69
Lane et al.^20^	0.65	0.33	0.64	0.62	0.72	0.29	0.71	0.7
Wegner et al.^21^	0.67	0.25	0.62	0.63	0.75	0.32	0.7	0.7

### Evaluation of the DeepPhase model performance on ES, ED and non-ESED cardiac phases

We evaluated the model performance in this experiment when trained and tested over ES, ED and Non-ESED frames. We used the mean value of 5-fold cross-validation for the results reported in this section.

#### ES and Non-ESED classification

The Table 3 compares the performance of eight different models, including DeepPhase, CNN proposed by Siddiqi^27^, VGG16, MobileNet, EfficientNet, ResNet, Lane et al.^20^, and Wegner et al.^21^ for ES and Non-ESED frames classification.

Among these models, DeepPhase achieved the highest AUC score of 0.8, indicating better classification performance than other models. DeepPhase also has the lowest Loss value of 0.15, indicating that it has the lowest difference between the predicted output and actual output. Additionally, DeepPhase has the highest Recall of 0.75, indicating that it was better at identifying positive samples in the dataset. Lane et al.^20^ and Wegner et al.^21^ achieved AUC scores of 0.71 and 0.76, respectively. Lane et al.^28^ has the precision of 0.72, indicating that it performed better at identifying true positives among the predicted positives than Wegner et al.^21^. The remaining models, including CNN proposed by Siddiqi^27^, VGG16, MobileNet, EfficientNet, and ResNet, has lower performance scores compared to DeepPhase, Lane et al.^20^, and Wegner et al.^21^.

#### ED and non-ESED classification

The Table 4 presents the performance of different models in identifying ED frames and Non-ESED frames, based on the evaluation metrics of AUC, Loss, Precision, and Recall. Among the models, DeepPhase achieved the highest AUC score of 0.85, indicating the best performance in distinguishing between positive and negative samples. It also has the lowest Loss value of 0.15, indicating the lowest difference between predicted and actual outputs. However, its recall score decreased to 0.72.

Wegner et al.^21^ has the AUC score of 0.81, and Lane et al.^20^ has the AUC score of 0.8. These models also has high Precision scores of 0.71 and 0.7, respectively, indicating that they performed well in identifying true positives among the predicted positives. EfficientNet also performed well in this dataset, achieving an AUC score of 0.76 and Precision score of 0.7. CNN proposed by Siddiqi^27^, VGG16, and ResNet has AUC scores ranging from 0.68 to 0.71, with Recall scores ranging from 0.65 to 0.69.

#### ED, ES and Non-ESED classification

In Table 5 shows the performance of competing approaches for ES, ED, and Non-ESED classes. DeepPhase outperformed all other models with an AUC of 0.79 and a precision of 0.76. Siddiqi’s model^27^ has the lowest AUC and precision. Among the other models, Wegner et al.’s^21^ model performed better than Lane et al.’s model^20^. EfficientNet and ResNet have comparable performance while MobileNet and VGG16 models have lower AUC and precision than other models.

One of the key contributions of the proposed approach is that it provides a solution to the imbalanced classification problem faced by Lane et al.^20^ for cardiac phase detection. The imbalanced class problem would arise because Non-ESED class would be greatly over-represented. Another study^30^ proposed a binary classification approach, which labeled all frames in the diastole phase as 1 and systole phase as 0. However, this approach can overlook high-level spatial and temporal markers and crucial physiological differences throughout the entire cardiac cycle, leading to lower accuracy and suboptimal model performance. By randomly selecting Non-ESED frames from the rest of the frames and ensuring that each video has an ES, ED, and Non-ESED frame, our approach provides a more balanced and unbiased dataset that can capture the complexity and variability of the cardiac cycle more accurately. Therefore, this research work represents a significant improvement over the previous study and can potentially enhance the accuracy and reliability of cardiac phase detection models

In the results section, it is evident that the DeepPhase model has several advantages over previously proposed regression models^6,20^ in the context of detecting end-systolic (ES) and end-diastolic (ED) frames in echocardiography videos. As noted in^26^, there can be a lot of variation in the images due to differences in patient anatomy and imaging conditions. The DeepPhase classification model can better handle this variability by focusing on the most relevant features for each class. Additionally, the DeepPhase model is more scalable than a regression model, as it can be trained on a smaller dataset, and it may be better at generalizing to new data than a regression model, as it focuses on the separation of classes rather than on fitting the data to a specific function^31,32^. These advantages are supported by the results presented in the paper, and they highlight the potential of the DeepPhase model in improving the accuracy and reliability of cardiac phase detection models.

Moreover, by randomly selecting three frames from each video, including ES, ED, and Non-ESED frames, we created a dataset that addressed the challenge of variable length videos. Specifically, this approach enabled us to train and test our model on a set of videos with different lengths while ensuring that each video contained the necessary frames to classify the cardiac phase accurately. This helped us overcome the limitations of previous studies^23,30^ that were constrained by fixed length videos containing only one cardiac cycle.

**Figure 11:**
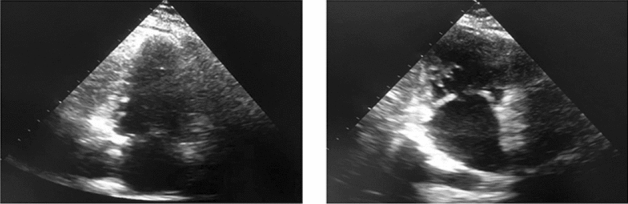
The misclassified ED frames from CardiacPhase dataset.

We asked the echocardiographer’s opinion about some of the images misclassified by the model, as shown in Fig. 11. The echocardiographer indicated that the manual classification of such images is also challenging for several reasons. One of the most common factors for the misclassification of ES and ED frames is the poor acoustic window in obese patients. The absence of a proper acoustic window during the echo process results in noisy images that are difficult to classify manually and by the model. In most poor-quality images, the mitral valve that separates LV and left atrium is not visible properly. According to the expert echocardiographer, to classify the following frames manually, an echocardiographer must know the ground realities, such as the weight and medical history of the patient and the actual measurements captured by the echo machine. This observation can help in future in proposing a fused model which does not classify based on images only but also takes into account the measurements and medical history of the patients.

### Ablation study

We conducted the following analysis as an ablation study to compare the performance of DeepPhase model with other traditional loss functions. As part of this study, we also performed binary classification for ES, ED and Non-ESED with and without cropped images.

#### Customized vs non-customized (i.e, traditional) loss functions

**Figure 12:**
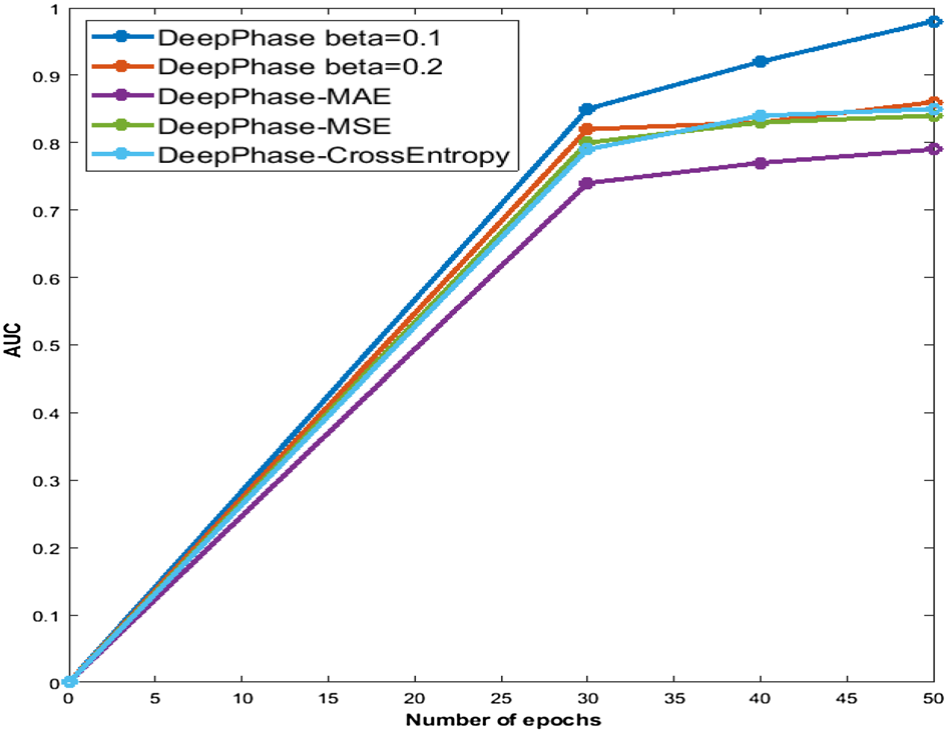
The training AUC of DeepPhase on epochs 30, 40 and 50.

In this study, we show the effectiveness of customizing the loss function. The training AUC of DeepPhase with customized and traditional loss functions at epochs 30, 40, and 50 are given in Fig. 12, and the testing AUC of each model is presented in Table 6.

In the experiment, we explored the best results on CAMUS when the $\beta$ value was set to 0.1, i.e., testing AUC = 0.96 and testing loss = 0.08. The highest AUC among all approaches for the CardiacPhase dataset achieved by DeepPhase is 0.82 with $\beta$ = 0.2. This high AUC was achieved despite CAMUS and CardiacPhase datasets having different frame quality and distributions. This demonstrates the flexibility of the DeepPhase model and custom loss function. The DeepPhase with MSE, L1 Regularizer, and L2 Regularizer all perform better than the DeepPhase with CrossEntropy and MAE, but still do not perform as well as DeepPhase with $\beta=0.1$ and $\beta=0.2$ on both datasets.

#### Image cropping vs non-cropping

In this study, we report the effect of image cropping by comparing competing models performance on uncropped images. We evaluated the model performance in this experiment when trained and tested over (1) ES and Non-ESED (2) ED and Non-ESED classes and (3) ES, ED and Non-ESED. We used the mean value of 5-fold cross-validation for the results reported in this section. Table 7 shows the model’s performance for the ES and Non-ESED classification experiment. The highest AUC achieved is 0.71 for ES and Non-ESED classes before applying the cropping technique, which is explained in “Methodology”. After we cropped the images, the competing models’ performance improved. The performance of the DeepPhase model enhanced from 0.71 to 0.75 with a loss of 0.2 and 0.15, respectively. ResNet takes another noticeable leap in performance from 0.6 to 0.71.

Table 8 shows the performance of ED and Non-ESED classification. Our cropping technique drastically improved the performance of the DeepPhase model from 0.7 to 0.84. The AUC for the ED phase is overall better than the ES phase for all the models. We can conclude from these results that the ED phase is easily differentiated from the Non-ESED phase compared to the ES phase. This cropping technique is not specific to this problem and can be applied to other medical images and images from different domains to improve the network’s performance. This cropping technique can also help to reduce the training and testing time.

Looking at the Table 9, we can see that the performance of DeepPhase model improved significantly on the cropped dataset for Es, ED and Non-ESED classification. The AUC score increased from 0.72 on the uncropped dataset to 0.79 on the cropped dataset, indicating that the model’s ability to distinguish between positive and negative samples improved with the cropped images.

Furthermore, the precision score increased from 0.63 to 0.76, and the recall score increased from 0.7 to 0.78 on the cropped dataset, indicating that the model’s ability to correctly identify positive samples improved with the cropped images. we can also see that the performance of other models improved on the cropped dataset, but the magnitude of improvement varies among the models.

## Conclusion

In this paper, we propose a method to directly solve the ES, ED, and Non-ESED phase detection problem without the need for LV segmentation. The DeepPhase model successfully classifies frames from unseen data, and experimental results confirm that the proposed MSE-based loss function outperforms other standard loss functions. To mitigate the lack of publicly available data for echocardiography, we introduce a dataset of 70 images, which we use as a test set in our experiments. To test the generalizability of the DeepPhase model, we also experiment with the apical four chamber view and obtain comparable results. Additionally, we introduce a cropping technique that can be useful for other medical images. However, to further improve the results presented in this paper, we acknowledge the need for a larger dataset to train the DeepPhase model. Therefore, in future work, we plan to develop a larger dataset for echocardiography views and train the DeepPhase model using transfer learning techniques to enhance its performance.

## Data Availability

The data presented in this study are available on request from the first authors. An email can be sent to 201990108@uaeu.ac.ae for data access.
