# Author Correction: Cardiac phase detection in echocardiography using convolutional neural networks

**DOI:** 10.1038/s41598-023-37788-5

**Published:** 2023-06-30

**Authors:** Moomal Farhad, Mohammad Mehedy Masud, Azam Beg, Amir Ahmad, Luai A. Ahmed, Sehar Memon

**Affiliations:** 1 College of Information Technology, United Arab Emirates University, P.O. Box 15551, Al Ain, United Arab Emirates; 2 Institute of Public Health, College of Medicine and Health Sciences, United Arab Emirates University, Al Ain, United Arab Emirates; 3 Indus Medical College, Hyderabad, Pakistan

Correction to: *Scientific Reports* https://doi.org/10.1038/s41598-023-36047-x, published online 01 June 2023

The original version of this Article contained an error in the spelling of the author Luai A. Ahmed, which was incorrectly given as Luai Ahmed.

The original Article has been corrected.

